# Effects of risperidone/paliperidone versus placebo on cognitive functioning over the first 6 months of treatment for psychotic disorder: secondary analysis of a triple-blind randomised clinical trial

**DOI:** 10.1038/s41398-023-02501-7

**Published:** 2023-06-10

**Authors:** Kelly Allott, Hok Pan Yuen, Lara Baldwin, Brian O’Donoghue, Alex Fornito, Sidhant Chopra, Barnaby Nelson, Jessica Graham, Melissa J. Kerr, Tina-Marie Proffitt, Aswin Ratheesh, Mario Alvarez-Jimenez, Susy Harrigan, Ellie Brown, Andrew D. Thompson, Christos Pantelis, Michael Berk, Patrick D. McGorry, Shona M. Francey, Stephen J. Wood

**Affiliations:** 1 Orygen, Parkville, VIC Australia; 2 Centre for Youth Mental Health, The University of Melbourne, Parkville, VIC Australia; 3 Department of Psychiatry, University College Dublin, Belfield, Ireland; 4 Turner Institute for Brain and Mental Health, School of Psychological Sciences, Monash University, Clayton, VIC Australia; 5 Monash Biomedical Imaging, Monash University, Clayton, VIC Australia; 6 Department of Psychology, Yale University, New Haven, CT USA; 7 Department of Social Work, School of Primary and Allied Health Care, Monash University, Melbourne, VIC Australia; 8 Centre for Mental Health, Melbourne School of Global and Population Health, The University of Melbourne, Parkville, VIC Australia; 9 Division of Mental Health and Wellbeing, Warwick Medical School, University of Warwick, Warwick, UK; 10 Melbourne Neuropsychiatry Centre, Department of Psychiatry, The University of Melbourne, Parkville, VIC Australia; 11 Florey Institute of Neuroscience and Mental Health, The University of Melbourne, Parkville, VIC Australia; 12 NorthWestern Mental Health, Western Hospital Sunshine, St Albans, VIC Australia; 13 Deakin University, IMPACT – the Institute for Mental and Physical Health and Clinical Translation, School of Medicine, Barwon Health, Geelong, VIC Australia; 14 School of Psychology, University of Birmingham, Edgbaston, UK

**Keywords:** Clinical pharmacology, Schizophrenia

## Abstract

The drivers of cognitive change following first-episode psychosis remain poorly understood. Evidence regarding the role of antipsychotic medication is primarily based on naturalistic studies or clinical trials without a placebo arm, making it difficult to disentangle illness from medication effects. A secondary analysis of a randomised, triple-blind, placebo-controlled trial, where antipsychotic-naive patients with first-episode psychotic disorder were allocated to receive risperidone/paliperidone or matched placebo plus intensive psychosocial therapy for 6 months was conducted. A healthy control group was also recruited. A cognitive battery was administered at baseline and 6 months. Intention-to-treat analysis involved 76 patients (antipsychotic medication group: 37; 18.6_Mage_ [2.9] years; 21 women; placebo group: 39; 18.3_Mage_ [2.7]; 22 women); and 42 healthy controls (19.2_Mage_ [3.0] years; 28 women). Cognitive performance predominantly remained stable (working memory, verbal fluency) or improved (attention, processing speed, cognitive control), with no group-by-time interaction evident. However, a significant group-by-time interaction was observed for immediate recall (*p* = 0.023), verbal learning (*p* = 0.024) and delayed recall (*p* = 0.005). The medication group declined whereas the placebo group improved on each measure (immediate recall: *p* = 0.024; *η*_p_^2^ = 0.062; verbal learning: *p* = 0.015; *η*_p_^2^ = 0.072 both medium effects; delayed recall: *p* = 0.001; *η*_p_^2^ = 0.123 large effect). The rate of change for the placebo and healthy control groups was similar. Per protocol analysis (placebo *n* = 16, medication *n* = 11) produced similar findings. Risperidone/paliperidone may worsen verbal learning and memory in the early months of psychosis treatment. Replication of this finding and examination of various antipsychotic agents are needed in confirmatory trials. Antipsychotic effects should be considered in longitudinal studies of cognition in psychosis.

Trial registration: Australian New Zealand Clinical Trials Registry (http://www.anzctr.org.au/; ACTRN12607000608460).

## Introduction

Widespread cognitive impairments are a core feature of psychotic disorders, being present prior to [1] and during the first-episode of psychosis (FEP) [2, 3]. In both antipsychotic-naive and antipsychotic-exposed FEP patients, medium-to-large impairments are routinely observed. The most severe impairments are in verbal learning and memory, processing speed and working memory [2, 3]. Generally, cognitive impairments remain fairly stable [4, 5], although recent longitudinal studies have shown decline in specific domains years after FEP [6–8].

The causes of cognitive change in psychosis remain poorly understood. One contentious factor is the role of antipsychotic medication. It is unclear whether antipsychotics ameliorate, exacerbate, or have negligible effects on cognitive impairments and whether effects differ by medication type. Clinical trials investigating the cognitive effects of atypical antipsychotics in first-episode or recent-onset schizophrenia-spectrum samples have documented mild cognitive improvements over a 3-to-6-month period (regardless of antipsychotic type) [9–13]. These improvements were typically modest, with further examination suggesting improvements were mostly due to test practice [9, 13]. Small improvements in cognitive functioning following antipsychotic treatment may also occur due to symptom improvement [10]. It is unknown whether cognitive improvements would also be observed with symptom improvement *in the absence of* antipsychotic medication. Most trials examining the cognitive effects of antipsychotics have been head-to-head (e.g., typical vs. atypical or atypical comparisons) without a placebo or healthy control group [9, 10, 12]. Thus, the effect of antipsychotics on cognitive functioning could not be clearly disentangled from the illness itself. Older trials were also compromised by inequitable dosing (i.e., first-generation prescribed at higher equivalent dose compared to second-generation) potentially inflating the beneficial effects of second-generation antipsychotics on cognition [14]. Given cognitive functioning is among the most robust predictors of functional recovery following FEP [15, 16], it is critical to understand what effect antipsychotic treatment has on cognition.

Most cognitive impairment seems to occur prior to or during the development of full-threshold psychotic disorder [17, 18]. However, because antipsychotics are usually the first-line treatment for all psychotic disorders, understanding the ongoing course of cognitive functioning post-treatment is complicated. Specifically, do antipsychotics prevent or exacerbate further cognitive decline or lag? Do their effects differ by cognitive domain? One early randomised placebo-controlled trial involving people with acute ‘functional psychosis’ showed that, at 2.5-year follow-up the cognitive functioning of those with an initial 4-week non-medication period (placebo) was no different from those who were immediately prescribed antipsychotics [19]. In other words, a 4-week delay in introducing antipsychotic medication did not result in long-term adverse cognitive effects. Meta-analyses of the relationship between duration of untreated psychosis (DUP), usually defined as the time between psychotic symptom onset and adequate treatment with antipsychotic medication, and cognitive functioning suggest DUP is not associated with cognitive performance [20, 21]. Moreover, naturalistic studies have shown higher cumulative use of antipsychotics is associated with poorer cognitive functioning [22–24], but these findings may be confounded by illness severity and cohort effects.

The primary aim of this study was to disentangle the effects of psychotic illness from those of antipsychotic medication on cognition over the first 6 months of treatment for a first-episode psychotic disorder. We analysed cognition data collected within a randomised, triple-blind placebo-controlled trial [25]. A healthy control group was also recruited to account for typical cognitive development and practice effects. The cognition outcomes reported here were a pre-planned secondary analysis of the Staged Treatment and Acceptability Guidelines in Early Psychosis (STAGES) trial [26], where we previously reported that the placebo group had comparable functional outcomes (primary outcome) and clinical outcomes to the group receiving antipsychotic medication [25], but showed different trajectories of brain volume and function [27, 28].

## Methods and materials

### Study design

STAGES was a randomised, triple-blind, placebo-controlled trial comparing the effects of antipsychotic medication plus CBCM (medication group) with placebo plus CBCM (placebo group) in FEP [25, 26]. FEP participants were randomised 1:1, stratified by sex and DUP, within six permuted blocks. DUP was included as a three-level factor (0–30, 31–90, >90 days). Clinicians, patients, and assessors remained blind to treatment allocation throughout the trial. The active treatment phase was 6 months, with assessments conducted at baseline (prior to treatment allocation) and 6 months. To account for practice effects, a healthy control group also completed baseline and 6-month assessments. The Melbourne Health Human Research Ethics Committee approved the study (MH-HREC: #2007:616). Trial registration was with the Australian New Zealand Clinical Trials Registry (ACTRN12607000608460).

### Participants

FEP patients were aged 15–25 years and presented for treatment at the Early Psychosis Prevention and Intervention Centre, a specialist public early psychosis programme in Melbourne’s northern-western suburbs, Australia. Participants met criteria for a DSM-IV psychotic disorder, including schizophrenia, schizophreniform disorder, schizoaffective disorder, delusional disorder, depression with psychosis, substance-induced psychotic disorder, or psychosis NOS, confirmed using the Structured Clinical Interview for DSM-IV (SCID-IV) for Axis I disorders. Inclusion criteria were: ability to provide informed consent; English language comprehension; DUP < 6 months; low past exposure to antipsychotic medication (<7 days or lifetime maximum 1750mg chlorpromazine equivalent); no previous lithium/anticonvulsant treatment; and to minimise risk: living in stable accommodation; low risk to self/others [25, 26].

Sex- and age-matched healthy controls were recruited via flyers, word-of-mouth, and social media advertisements. Inclusion criteria for healthy controls were aged 15–25 years and English language comprehension. Exclusion criteria included: history of psychotic disorder or any current mental disorder (screened using the SCID-IV Research Non-Patient Edition); head injury or neurological disorder; and studied psychology at university (to mitigate cognitive test exposure). All participants gave written informed consent, including parent/guardian consent for participants <18 years. All participants were reimbursed $50AUD per assessment.

### Treatment

FEP patients received weekly cognitive behavioural case management, a manualised formulation-based psychological and psychoeducation treatment for early psychosis [25]. Patients also saw a medical doctor weekly and received close monitoring, family therapy, vocational support, and 24-hour crisis support as required. Patients randomised to the medication group received either 1 mg risperidone (*n* = 33) or 3 mg paliperidone (*n* = 4), depending on the availability of medication and matched placebo [25]. The starting dose was titrated according to clinical response by the blinded study doctor. The same procedure was followed for the placebo group; allocated patients received a placebo pill that was identical to the active medication in taste, appearance, and packaging. To ensure participant safety, strict discontinuation criteria were applied in situations of insufficient clinical improvement, increased risk to self/others or worsening mental state/functioning and an alternative open-label antipsychotic medication was offered [26].

### Cognitive battery

The Information and Picture Completion subtests of the Wechsler Adult Intelligence Scale–Third Edition (WAIS-III) were administered at baseline to estimate current IQ. The repeated cognitive battery included measures of cognitive domains known to be most affected in psychotic disorders [3], including attention (WAIS-III Digit Span: forward), working memory (WAIS-III Digit Span: backward), processing speed (WAIS-III Digit Symbol-Coding), processing speed and inhibition (Golden Stroop: word, colour, colour-word subtests), verbal fluency (Controlled Oral Word Association Test, animal fluency), and verbal learning and memory (Paired-Associate Learning). All cognitive tasks are standard tasks [29] except for the Paired-Associate Learning task, described in more detail below. Raw scores were used in the analysis.

The verbal Paired-Associate Learning task used in the current study is a variant of a novel verbal associative learning task and was chosen because it has previously shown to be sensitive to medial temporal lobe dysfunction and to load minimally on attention and working memory [30]. This paired-associate learning paradigm uses arbitrary word pairs as opposed to words that are semantically related. Each trial included eight word pairs: four concrete word pairs (i.e., with high imageability, e.g., horse-forest) and four abstract word pairs (i.e., with low imageability, e.g., motion-honest). Imageability has been shown to be sensitive for assessing verbal learning, where durable memories are formed for highly imageable words in both healthy controls and neurological patients [30, 31]. The first part of the task is akin to a list learning paradigm, where the participant is presented with the eight words that form the first half of each word pair and asked to recall as many words as they can (List A). This is repeated a further two times. Then, List B is presented (i.e., the eight words that form the second part of each word pair) and learnt over three trials. Next, the assessor conducts the paired-associate learning part of the task, and reads the eight word pairs (i.e., each word pair is comprised of one word from List A and one word from List B) to the participant. After which the participant is presented with the first word and is asked to provide the second word that goes with it (i.e., cued recall). This is repeated a further two times, resulting in three paired-associate learning trials. The concrete and abstract pairs were presented in different orders for each trial. After a 20-minute delay, the participant again completed cued-recall, where they were given the first word and had to provide the second word of the previously learned pair. In the current study, the primary outcome variables from this task were Trial 1 of paired-associate learning (immediate recall: score out of 8), paired-associate learning (Trials 1–3 total; score out of 24) and delayed cued recall (score out of 8). Alternate forms of the task were used at baseline and 6 months.

### Statistical analysis

Baseline comparison of the three groups was conducted using analysis of variance, followed by pairwise comparison using Fisher’s least significant difference test. To assess rate of change in cognitive performance, group comparisons on each cognitive outcome were conducted using linear mixed-effects (LME) modelling analysis with random effects for intercept and slope and group-by-time interaction. Time was expressed as number of weeks from baseline to follow-up. The LME analysis included all cases with data on at least one timepoint (of 118 total participants, 84 had cognition data at both timepoints, and 34 only had data at baseline). Significant group-by-time interactions were followed by pairwise comparisons with effect size indexed using partial eta squared (*η*_p_^2^), indicating the proportion of variance explained by a given term in a model after accounting for variance explained by the other terms in the model (*η*_p_^2^ = 0.01 is considered small, 0.06 medium and 0.14 a large effect [32]). In accordance with CONSORT recommendations [33], a per protocol LME analysis was also performed, i.e., only including participants who remained on their allocated trial medication (placebo/medication) throughout the 6-month trial. A significance level of *p* < 0.05 was used (two-sided). Adjustment for multiple testing was not applied as this analysis was considered exploratory (not confirmatory) and was not powered for the cognition outcomes. In this scenario, priority is given to minimising type II error and generating evidence to inform future hypothesis-driven studies [34, 35]. The analysis was conducted using the *nlme* and *effectsize R* packages [32, 36].

## Results

**Fig. 1:**
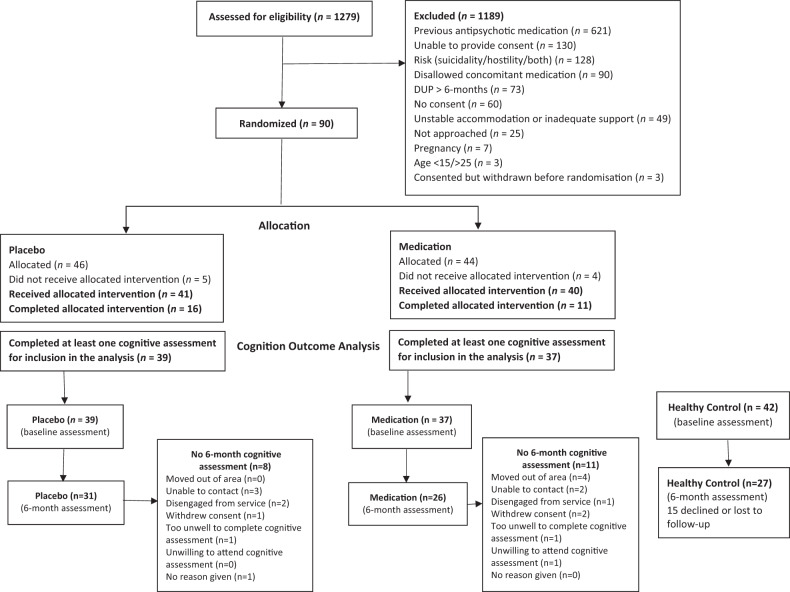
A diagram of participant flow into the study.

Figure 1 shows participant flow into the study. The placebo and medication groups did not significantly differ in number of missing 6-month assessments (*p* = 0.431) and there were few baseline differences between those who did and did not complete the 6-month assessment (Table S1).

The three groups were well matched on age and sex (Table 1). The placebo and medication groups were comparable on all baseline variables, including cognition. As expected, healthy controls had a significantly higher mean education, IQ, social and occupational functioning, and lower symptomatology than the FEP groups. Healthy controls also performed better than the FEP groups at baseline on all cognitive measures (Table S2).

**Table 1:** Participant characteristics at baseline.

	First-episode psychosis		Healthy control (*N* = 42)
	Placebo (*N* = 39)	Medication (*N* = 37)	
Age, years (SD)	18.3 (2.7)	18.6 (2.9)	19.2 (3.0)
Women, *N* (%)	22 (56.4%)	21 (56.8%)	28 (66.7%)
Education, years (SD)	11.8 (1.8)	12.0 (3.1)	13.4 (2.2)
Estimated IQ	89.4 (14.1)	91.8 (14.3)	107.6 (10.0)
BPRS Total, mean (SD)	58.7 (9.1)	57.5 (10.1)	31.6 (3.6)
BPRS Psychotic, mean (SD)	15.0 (3.1)	13.9 (3.7)	4.1 (0.3)
SOFAS, mean (SD)	53.8 (13.1)	53.3 (10.7)	80.5 (8.9)
SANS Total, mean (SD)	26.8 (15.1)	25.9 (15.0)	–
DUP, *N* (%)			
0–30 days	6 (15.4%)	6 (16.2%)	–
31–90 days	14 (35.9%)	12 (32.4%)	–
>90 days	19 (48.7%)	19 (51.4%)	–
Psychotic diagnosis, *N* (%)			
Major depression with psychosis	7 (17.9%)	8 (21.6%)	–
Schizophreniform disorder	6 (15.4%)	8 (21.6%)	–
Psychotic disorder NOS	12 (30.8%)	8 (21.6%)	–
Schizophrenia	7 (17.9%)	5 (13.6%)	–
Substance-induced psychotic disorder	6 (15.4%)	4 (10.8%)	–
Delusional disorder	1 (2.6%)	4 (10.8%)	–

*BPRS* Brief Psychiatric Rating Scale, *SOFAS* Social and Occupational Functioning Assessment Scale, *SANS* Scale for the Assessment of Negative Symptoms, *DUP* duration of untreated psychosis, *NOS* not otherwise specified.

### Primary analysis

**Fig. 2:**
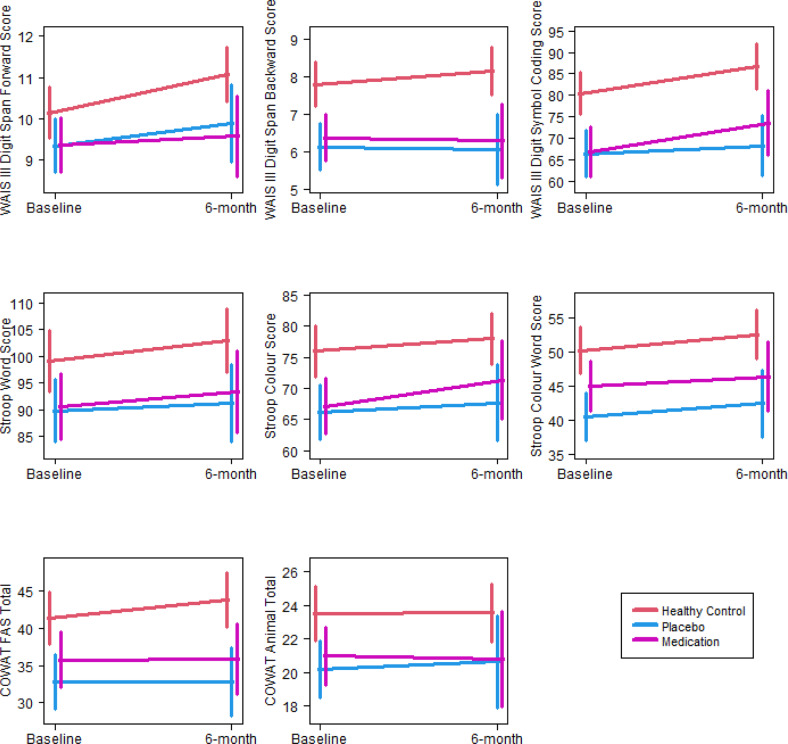
Plots of estimated trends for cognitive measures with no significant group × time interaction from baseline to 6 months and associated 95% confidence intervals for the means at endpoints. Each panel represents a different cognitive test score, with the test score shown on the *y*-axis and the timepoint shown on the *x*-axis.

The three groups were compared on rate of change in cognitive functioning using LME analyses (Table 2). A significant effect of time was observed on tasks of attention, processing speed and cognitive control (i.e., Digit Span: forward, Digit Symbol-Coding, Stroop: colour, word, colour-word); the estimated rates of change were all positive, indicating there was a significant improvement from baseline to 6 months on these measures regardless of group. Stable cognitive performance was observed in working memory (i.e., Digit Span: backward) and verbal fluency (letter/animal). There were no group-by-time interactions on any of these measures (Fig. 2).

**Table 2:** Results of LME analysis comparing the three groups on the rate of cognitive change from baseline to 6 months.

	*p* values			Estimated rate of change		*N*
	Group × time interaction	Group	Time	Coefficient	Standard error	
Digit Span: forward	0.180	**0.029**	**<0.001**	0.023	0.006	118
Digit Span: backward	0.507	**<0.001**	0.654	0.003	0.006	118
Digit Symbol-Coding	0.126	**<0.001**	**<0.001**	0.185	0.043	107
Stroop: words	0.605	**0.026**	**0.015**	0.107	0.039	117
Stroop: colours	0.452	**0.001**	**0.006**	0.096	0.035	117
Stroop: colour-word	0.879	**<0.001**	**0.016**	0.073	0.030	117
Letter fluency	0.295	**0.002**	0.152	0.036	0.025	118
Animal fluency	0.889	**0.011**	0.853	0.003	0.019	118
Immediate recall (Trial 1)	**0.023**	**<0.001**	0.138	0.014	0.009	118
Verbal learning (Trials 1–3)	**0.024**	**<0.001**	0.457	0.014	0.020	118
Delayed recall	**0.005**	**<0.001**	0.772	0.0004	0.007	117

Note. The *p* values for ‘Group × time interaction’ indicates if the three groups differ in terms of the rate of change from baseline to 6-month assessment. The *p* values for ‘Group’ indicate the significance of the group main effect, i.e., the overall difference between the group means. For those measures with no significant group × time interaction, this is a similar test to an ANOVA of the baseline values. The *p* values for ‘Time’ indicate the significance of the overall rate of change assuming there is no group × time interaction, i.e., if all the groups have the same rate of change. When there is significant group × time interaction (i.e., when there is significant difference in rate of change between the groups), then ‘Time’ may not be meaningful.

Bold values denote significant *p* values.

**Fig. 3:**
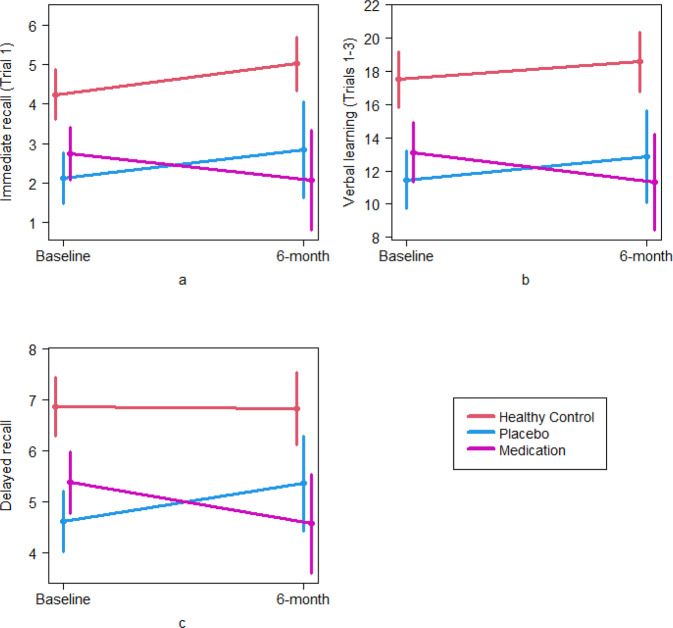
Plots of estimated trends for significant group × time interaction for verbal paired associate learning and memory from baseline to 6 months and associated 95% confidence intervals for the means at the endpoints. Each panel represents a different cognitive test score (**a** immediate recall; **b** verbal learning; and **c** delayed recall), with the test score shown on the *y*-axis and the timepoint shown on the *x*-axis.

Significant group-by-time interactions were found on the verbal learning and memory task, including immediate recall (*p* = 0.023), verbal learning (*p* = 0.024) and delayed recall (*p* = 0.005) (Table 2). Figure 3 plots estimated trends for the three groups from baseline to 6 months on these measures. Figure 3a illustrates the difference in rate of change in immediate recall, where the healthy control and placebo groups show an improvement, and the medication group shows a deterioration. Similar patterns can be observed in Fig. 3b, c. Pairwise comparisons showed a significant decline in the medication group compared to the healthy control (*p* = 0.013; *η*_p_^2^ = 0.074 medium effect) and placebo (*p* = 0.024; *η*_p_^2^ = 0.062 medium effect) groups for immediate recall, as well as verbal learning (healthy control vs. medication *p* = 0.022; *η*_p_^2^ = 0.064 medium effect; placebo vs. medication *p* = 0.015; *η*_p_^2^ = 0.072 medium effect). For delayed recall, the medication group again showed significant decline compared to the placebo group (*p* = 0.001; *η*_p_^2^ = 0.123 large effect); however, neither group showed a significant difference in rate of change compared to healthy controls (healthy control vs. placebo *p* = 0.072; *η*_p_^2^ = 0.040 medium effect; healthy control vs. medication *p* = 0.090; *η*_p_^2^ = 0.036 small effect).

### Per protocol analysis

During the 6-month trial the mean cumulative dose of antipsychotics (olanzapine equivalent mg) was 858.2 (SD = 512.1; range 4–2105) in the medication group and 314.5 (SD = 490.0; range 0–1773) in the placebo group, a significant difference (*p* < 0.001). Nineteen patients from the placebo group and 26 from the medication group stopped their allocated medication (placebo or risperidone/paliperidone) during the 6-month trial and many commenced a different medication (Table S3). Reasons for stopping were similar between groups [25] (Fig. S1).

To test the reliability of the intention-to-treat findings, a per protocol analysis was conducted including only trial completers (placebo *n* = 16, medication *n* = 11, healthy controls *n* = 42; Table S4; Fig. S2). Results remained similar, with significant group-by-time interactions on the verbal learning and memory task, specifically verbal learning (*p* = 0.025) and delayed recall (*p* < 0.001); with immediate recall just non-significant (*p* = 0.051). Pairwise comparisons showed that all three groups differed from each other in rate of change for delayed recall (healthy control vs. placebo *p* = 0.004; *η*_p_^2^ = 0.154; healthy control vs. medication *p* = 0.012; *η*_p_^2^ = 0.123; placebo vs. medication *p* < 0.001; *η*_p_^2^ = 0.297; all large effects). There was a significant difference in the rate of change between the medication group and the other two groups for immediate recall (healthy control vs. medication *p* = 0.030; *η*_p_^2^ = 0.093; placebo vs. medication *p* = 0.029; *η*_p_^2^ = 0.093; medium–large effects) and verbal learning (healthy control vs. medication *p* = 0.018; *η*_p_^2^ = 0.109; placebo vs. medication *p* = 0.010; *η*_p_^2^ = 0.129; medium–large effects). Additionally, a group-by-time interaction was seen for digit symbol-coding (*p* = 0.016), where the healthy control group improved, but the medication and placebo groups remained stable (healthy control vs. placebo *p* = 0.028; *η*_p_^2^ = 0.099; healthy control vs. medication *p* = 0.019; *η*_p_^2^ = 0.111; medium–large effects).

## Discussion

This pre-planned secondary analysis of a triple-blind, placebo-controlled, randomised clinical trial, with a demographically matched healthy control group, enabled us to determine the effects of psychotic disorder, antipsychotic medication, and typical changes on cognitive performance over 6 months. The prospective randomised design ensured the medication and placebo groups did not significantly differ at baseline on any cognitive measure, length of DUP, or other illness variables. Two key findings emerged. First, change in cognitive functioning was similar across the placebo, medication, and healthy control groups in most cognitive domains. This suggests that the cognitive improvement or stability observed was not specifically associated with the effects of medication or illness, as the same pattern of change was also seen in healthy controls with no history of psychosis or antipsychotic exposure. The second key finding was that allocation to the risperidone/paliperidone over 6 months was associated with a decline in verbal learning and memory. In contrast, patients who received placebo improved in verbal learning and memory at a similar rate to healthy controls. The effect sizes were medium to large. These findings were broadly similar when only trial completers were included in the analysis.

A significant implication of the findings is that partially or completely withholding antipsychotics is not harmful to cognitive functioning and may be specifically beneficial for verbal learning and memory in the early course of treatment. Experimentally lengthening the DUP (in the placebo group) was not associated with worsening of cognition or a slower rate of improvement in any domain measured. This finding is consistent with previous meta-analyses showing negligible associations between DUP and cognition [20, 21]. On most measures, the change in cognition was similar across the placebo, medication, and healthy control groups, suggesting improvements over 6 months are likely explained by typical development or practice effects [13] and not symptom improvement or medication effects.

A second key implication of the findings is that risperidone/paliperidone may worsen verbal learning and memory at least for some young FEP patients not highly suicidal or hostile and with short DUP. This finding contrasts with earlier randomised head-to-head trials showing mildly improved cognitive functioning, including verbal learning and memory, following 3–6 months of treatment with second-generation antipsychotics, including risperidone [9, 10, 12, 13]. Differences between previous studies and our study may account for this. First, previous studies did not include a placebo arm [13]. Second, participants in previous studies were older on average [9, 10, 12]. Third, we enrolled patients diagnosed with *any* psychotic disorder, whereas only people diagnosed with schizophrenia-spectrum disorders were included in previous trials. Perhaps most importantly, all previous trials employed wordlist learning memory tasks comprising nouns that can be imagined and semantically-encoded [9, 10, 12, 13] (one study also included a story memory task [13]). We used an associate learning task, involving concrete and *abstract* word-pairs, where the latter are more difficult to imagine and encode [31] (see Table S5); therefore, our task was arguably more sensitive than those used previously [29, 37]. We tested this proposition in a post hoc LME analysis of the wordlist learning component of the task and found no significant group × time interaction (Table S6). Thus, risperidone/paliperidone treatment for some young people with early course psychosis may impair novel effortful associate learning and memory.

These effects of risperidone/paliperidone on learning and memory may arise because of their strong antagonism at dopamine (D_2_) receptors [38]. D_2_ receptors are prominently expressed in brain regions involved in learning and memory, particularly the striatum, prefrontal cortex, and hippocampus [39, 40]. Previous research shows significant negative correlations between risperidone-associated extrastriatal D_2/3_ occupancy and cognitive performance [41] and striatal D_2_ occupancy and subjective cognitive effects [42]. Given antipsychotics as a group differ in their off-target effects outside of dopamine (D_2_) antagonism and may differ in effects on cognition as well [43], our findings cannot be generalised to all antipsychotic types.

Side-effects such as sedation, movement disorders, blurred vision and amotivation may also arise in response to taking risperidone/paliperidone [44–47], all of which can impair cognitive functioning and performance on cognitive tasks [47]. This explanation seems unlikely in this study because we found no significant difference between the FEP groups in terms of antipsychotic side-effects (including cognitive and sedative effects) as rated by the study doctors [25]. It is worth noting that we did not assess subjective effects. Subjective side-effects as listed above may have affected cognitive capacity or cognitive effort on the verbal paired-associate learning task, which was the most effortful and difficult task within the cognitive battery. Future research should measure subjective medication effects as potential mechanisms of cognitive impairment (or improvement) in psychotic disorders.

An anticholinergic mechanism could also plausibly explain the observed decline in verbal learning and memory [48–50]; however, risperidone/paliperidone have a relatively low anticholinergic profile [51]. Furthermore, the doses prescribed here, while still considered therapeutic [25], were relatively low. Use of other medications, such as antidepressants and benzodiazepines also have low anticholinergic activity [51] and did not differ between groups [25]. The anticholinergic benztropine was prescribed PRN to three cases in the medication group, but they were excluded from the completer analysis. Thus, anticholinergic effects are an unlikely explanation for the findings.

The specificity of the negative effect of risperidone/paliperidone to learning and memory is notable given impairment in this domain is associated with poorer community/vocational and social functioning in early psychosis [15], and shows decline in longitudinal studies of FEP (including in paired-associate learning) [6–8]. The contribution of medication to cognitive decline cannot be accurately determined in these cohort studies, since cumulative antipsychotic exposure is confounded with illness severity; however the current findings suggest antipsychotic medication may play a role [52]. In support of this supposition, antipsychotic dose reduction has been associated with improved cognitive functioning (including learning and memory) in naturalistic longitudinal studies [22] and RCTs [53, 54] involving FEP and schizophrenia patients.

These cognition findings must be considered within the context of the full risk: benefit profile of withholding or prescribing antipsychotics in the early stages of psychosis treatment, including symptomatology, functioning, physical and metabolic health, brain health outcomes, in addition to patient-driven personal recovery goals [55]. The STAGES study is one of the few placebo-controlled studies to examine the risks and benefits of antipsychotic treatment in FEP [56]. We have previously reported that the placebo group had comparable functioning (primary outcome), symptomatology, quality of life, adverse events and physical and metabolic outcomes to the group receiving antipsychotic medication [25, 57]. We also showed an increase in right pallidum volume in the risperidone/paliperidone group and decline in the placebo group over the first three months of the trial, suggestive of a potentially early protective effect of antipsychotic medication [27]. However, increased connectivity was observed in different brain regions across the placebo and medication groups, suggesting psychosocial treatment alone can lead to improved brain function [28]. More high-quality clinical trials are needed to support evidence-based shared decision-making among clinicians, patients, and their caregivers.

It is important to acknowledge that, in addition to the small sample, this was a distinctive and highly selective FEP sample due to the inclusion/exclusion criteria, so findings may not generalise to all FEP individuals and certainly require replication in confirmatory trials. Nevertheless, the degree of baseline cognitive impairment in the sample was comparable to other FEP samples diagnosed with schizophrenia [3]. We were not able to report on medication adherence due to a large amount of missing adherence data. Another limitation is the high percentage of participants who did not complete the trial as planned. However, the per protocol analysis was mostly consistent with the primary analysis (apart from immediate recall), suggesting results are quite robust. Finally, while regression to the mean may be an explanation for the verbal learning and memory findings, this is unlikely because the differences between the placebo and medication groups at baseline were small and non-significant (Table S2) and randomisation controls for this effect across groups [58, 59].

In conclusion, the findings highlight the importance of accounting for the potential longitudinal cognitive effects of antipsychotic medication following FEP. Careful consideration of the risks and benefits of antipsychotic initiation and maintenance is critical and should occur within a shared decision-making framework. Indeed, a recent study showed that of various antipsychotic side effects, memory impairment had the strongest influence on the medication preferences of people with schizophrenia [60]. Future research must investigate class differences in the effects and mechanisms of action of antipsychotics on various domains of cognition. If this confirms what is reported here, then efforts to address cognitive impairment in FEP should consider medication effects, alongside a focus on developing novel agents and psychosocial treatments with pro-cognitive effects.

## Supplementary information

Supplementary material
