# Supplementary material for: Effects of risperidone/paliperidone versus placebo on cognitive functioning over the first 6 months of treatment for psychotic disorder: secondary analysis of a triple-blind randomised clinical trial
Source: Transl Psychiatry. 2023 Jun 10;13:199. doi: 10.1038/s41398-023-02501-7 (PMC10257667; doi:10.1038/s41398-023-02501-7)
Supplement: Supplementary file 1 — Supplementary material [file 41398_2023_2501_MOESM1_ESM.docx]

**Supplementary Table S1.** Comparisons of baseline variables within each study group between those with and without 6-month assessment

|  |  | Medication | | | | | Placebo | | | | HC | | | |
| --- | --- | --- | --- | --- | --- | --- | --- | --- | --- | --- | --- | --- | --- | --- |
|  | 6M ax? | Mean | SD | n | p-value* | | Mean | SD | n | p-value* | Mean | SD | n | p-value* |
| Age | No | 18.1 | 2.5 | 11 | 0.439 | | 17.6 | 2.8 | 9 | 0.424 | 18.5 | 1.9 | 15 | 0.210 |
|  | Yes | 18.8 | 3.0 | 26 |  | | 18.4 | 2.7 | 31 |  | 19.6 | 3.5 | 27 |  |
| Years of education | No | 12.1 | 2.3 | 10 | 0.856 | | 10.6 | 1.2 | 8 | 0.015 | 13.3 | 1.9 | 15 | 0.913 |
|  | Yes | 11.9 | 3.4 | 24 |  | | 12.1 | 1.9 | 28 |  | 13.4 | 2.4 | 27 |  |
| WAIS III FSIQ | No | 91.2 | 13.4 | 11 | 0.861 | | 79.9 | 9.6 | 8 | 0.012 | 104.8 | 10.7 | 15 | 0.195 |
|  | Yes | 92.1 | 15.0 | 24 |  | | 91.9 | 14.1 | 30 |  | 109.2 | 9.4 | 27 |  |
| BPRS Total | No | 53.7 | 9.8 | 11 | 0.147 | | 60.1 | 2.5 | 9 | 0.562 | 31.3 | 3.1 | 15 | 0.687 |
|  | Yes | 59.1 | 9.9 | 26 |  | | 58.2 | 3.3 | 31 |  | 31.8 | 3.9 | 27 |  |
| BPRS Psychotic Subscale | No | 14.1 | 3.9 | 11 | 0.861 | | 15.6 | 2.5 | 9 | 0.506 | 4.1 | 0.4 | 15 | 0.576 |
|  | Yes | 13.8 | 3.8 | 26 |  | | 14.9 | 3.3 | 31 |  | 4.1 | 0.3 | 27 |  |
| SOFAS | No | 54.4 | 11.6 | 11 | 0.707 | | 56.3 | 8.4 | 9 | 0.455 | 79.0 | 8.4 | 15 | 0.420 |
|  | Yes | 52.8 | 10.5 | 26 |  | | 53.5 | 14.2 | 31 |  | 81.3 | 9.3 | 27 |  |
| Digit span: forward | No | 8.9 | 2.3 | 11 | | 0.444 | 8.5 | 1.4 | 8 | 0.117 | 9.9 | 2.0 | 15 | 0.611 |
|  | Yes | 9.5 | 1.9 | 25 | |  | 9.6 | 2.4 | 31 |  | 10.3 | 1.9 | 27 |  |
| Digit span: backward | No | 6.6 | 1.6 | 11 | | 0.412 | 5.0 | 1.7 | 8 | 0.076 | 7.5 | 2.0 | 15 | 0.379 |
|  | Yes | 6.2 | 1.6 | 25 | |  | 6.4 | 2.5 | 31 |  | 8.0 | 1.9 | 27 |  |
| Digit Symbol Coding | No | 70.3 | 13.1 | 9 | | 0.411 | 55.3 | 14.6 | 7 | 0.053 | 82.7 | 13.4 | 15 | 0.476 |
|  | Yes | 65.5 | 17.3 | 21 | |  | 69.2 | 16.4 | 28 |  | 79.1 | 17.9 | 27 |  |
| Stroop: word | No | 89.9 | 25.7 | 10 | | 0.978 | 71.2 | 20.5 | 8 | 0.012 | 102.5 | 13.6 | 15 | 0.237 |
|  | Yes | 90.2 | 20.2 | 25 | |  | 95.4 | 18.9 | 30 |  | 97.2 | 13.9 | 27 |  |
| Stroop: colour | No | 67.3 | 17.1 | 10 | | 0.992 | 56.8 | 14.1 | 8 | 0.054 | 75.7 | 8.6 | 15 | 0.936 |
|  | Yes | 67.2 | 15.0 | 25 | |  | 68.7 | 12.0 | 30 |  | 76.0 | 15.8 | 27 |  |
| Stroop: colour-word | No | 46.0 | 13.6 | 10 | | 0.743 | 31.4 | 10.9 | 8 | 0.023 | 49.3 | 8.0 | 15 | 0.677 |
|  | Yes | 44.3 | 13.0 | 25 | |  | 42.7 | 9.5 | 30 |  | 50.6 | 12.4 | 27 |  |
| Letter fluency | No | 37.3 | 10.2 | 11 | | 0.498 | 26.6 | 7.5 | 8 | 0.055 | 38.7 | 11.1 | 15 | 0.270 |
|  | Yes | 34.6 | 12.4 | 25 | |  | 33.6 | 11.2 | 30 |  | 42.8 | 11.6 | 27 |  |
| Animal fluency | No | 21.0 | 4.9 | 11 | | 0.846 | 17.1 | 4.4 | 10 | 0.545 | 23.0 | 3.1 | 15 | 0.480 |
|  | Yes | 20.6 | 6.0 | 24 | |  | 20.8 | 7.3 | 8 |  | 23.9 | 4.6 | 27 |  |
| Immediate recall (Trial 1) | No | 2.8 | 2.4 | 11 | | 0.911 | 2.0 | 1.1 | 8 | 0.768 | 4.3 | 2.3 | 15 | 0.912 |
|  | Yes | 2.7 | 2.2 | 25 | |  | 2.2 | 2.1 | 31 |  | 4.2 | 2.3 | 27 |  |
| Verbal learning (Trial 1-3) | No | 12.6 | 6.3 | 11 | | 0.776 | 9.8 | 4.9 | 8 | 0.305 | 16.7 | 6.4 | 15 | 0.539 |
|  | Yes | 13.3 | 5.7 | 25 | |  | 11.9 | 6.0 | 31 |  | 17.9 | 4.4 | 27 |  |
| Delayed recall | No | 5.0 | 2.2 | 11 | 0.533 | | 4.7 | 1.5 | 7 | 0.888 | 6.4 | 2.0 | 15 | 0.235 |
|  | Yes | 5.5 | 1.8 | 25 |  | | 4.6 | 2.4 | 31 |  | 7.1 | 1.3 | 27 |  |

* T-test

|  | | Medication | | | | Placebo | | | | | | HC | | | | | |
| --- | --- | --- | --- | --- | --- | --- | --- | --- | --- | --- | --- | --- | --- | --- | --- | --- | --- |
|  | | 6-month ax? | | |  | 6-month ax? | | | | |  | 6-month ax? | | | | |  |
|  | | No (n=11) | Yes (n=26) | | p-value* | No (n=8) | | Yes (n=31) | | | p-value* | No (n=15) | | Yes (n=27) | | | p-value* |
| % Male | | 36.4 | 46.2 | | 0.723 | 37.5 | | 45.2 | | | 1.000 | 40.0 | | 29.6 | | | 0.516 |
| DUP | % 0-30 | 18.2 | 15.4 | | 0.895 | 12.5 | | 16.1 | | | 1.000 |  | |  | | |  |
|  | % 31-90 | 36.4 | 30.8 | |  | 35.7 | | 35.5 | | |  |  | |  | | |  |
|  | % >90 | 45.5 | 53.8 | |  | 50.0 | | 48.4 | | |  |  | |  | | |  |
|  | | | | Medication | | | | | | Placebo | | | | | |  |  |
|  | | | | 6-month ax? | | | | |  | 6-month ax? | | | | |  |  |  |
| Psychotic Disorder Diagnosis | | | | No (n=11) | | | Yes (n=26) | | p-value* | No (n=8) | | | Yes (n=31) | | p-value* |  |  |
| Delusional disorder | | | | 0 | | | 15.4 | | 0.105 | 0 | | | 3.2 | | 0.101 |  |  |
| Major depression with psychosis | | | | 9.1 | | | 26.9 | |  | 0 | | | 22.6 | |  |  |  |
| Psychosis NOS | | | | 45.5 | | | 11.5 | |  | 37.5 | | | 29.0 | |  |  |  |
| Schizophreniform disorder | | | | 9.1 | | | 26.9 | |  | 0 | | | 19.4 | |  |  |  |
| Substance induced psychotic disorder | | | | 18.8 | | | 4.5 | |  | 12.5 | | | 16.1 | |  |  |  |
| Schizophrenia | | | | 18.2 | | | 11.5 | |  | 50.0 | | | 9.7 | |  |  |  |

* Fisher’s exact test**Supplementary Table S2.** Summary statistics of the cognitive measures at baseline

|  | Group | Mean | SD | n |  | P-values for the comparison of the 3 groups* | | | | Effect sizes (Cohen’s *d*) | | |
| --- | --- | --- | --- | --- | --- | --- | --- | --- | --- | --- | --- | --- |
|  |  |  |  |  | F | Overall | HC vs PLACEBO | HC vs MEDICATION | PLACEBO vs MEDICATION | HC vs PLACEBO | HC vs MEDICATION | PLACEBO vs MEDICATION |
| Digit Span: Forward | HC | 10.1 | 1.9 | 42 | 2.03 | 0.136 | 0.089 | 0.085 | 0.957 | 0.38 | 0.39 | 0.01 |
|  | PLACEBO | 9.4 | 2.2 | 39 |  |  |  |  |  |  |  |  |
|  | MEDICATION | 9.3 | 2.0 | 36 |  |  |  |  |  |  |  |  |
| Digit Span: Backward | HC | 7.8 | 1.9 | 42 | 8.79 | <0.001 | <0.001 | 0.001 | 0.704 | 0.85 | 0.76 | 0.09 |
|  | PLACEBO | 6.1 | 2.4 | 39 |  |  |  |  |  |  |  |  |
|  | MEDICATION | 6.3 | 1.6 | 36 |  |  |  |  |  |  |  |  |
| Digit Symbol-Coding | HC | 80.4 | 16.4 | 42 | 8.94 | <0.001 | <0.001 | 0.001 | 0.897 | 0.85 | 0.82 | 0.03 |
|  | PLACEBO | 66.4 | 16.8 | 35 |  |  |  |  |  |  |  |  |
|  | MEDICATION | 66.9 | 16.1 | 30 |  |  |  |  |  |  |  |  |
| Stroop: word | HC | 99.1 | 13.8 | 42 | 2.93 | 0.058 | 0.041 | 0.040 | 0.959 | 0.46 | 0.48 | 0.01 |
|  | PLACEBO | 90.3 | 21.4 | 38 |  |  |  |  |  |  |  |  |
|  | MEDICATION | 90.1 | 21.5 | 35 |  |  |  |  |  |  |  |  |
| Stroop: colour | HC | 75.9 | 13.6 | 42 | 5.85 | 0.004 | 0.002 | 0.008 | 0.739 | 0.70 | 0.62 | 0.08 |
|  | PLACEBO | 66.2 | 13.2 | 38 |  |  |  |  |  |  |  |  |
|  | MEDICATION | 67.3 | 15.4 | 35 |  |  |  |  |  |  |  |  |
| Stroop: colour-word | HC | 50.1 | 11.0 | 42 | 7.25 | 0.001 | <0.001 | 0.047 | 0.099 | 0.85 | 0.46 | 0.39 |
|  | PLACEBO | 40.3 | 10.7 | 38 |  |  |  |  |  |  |  |  |
|  | MEDICATION | 44.8 | 13.0 | 35 |  |  |  |  |  |  |  |  |
| Letter fluency | HC | 41.4 | 11.5 | 42 | 6.91 | 0.001 | <0.001 | 0.022 | 0.215 | 0.82 | 0.53 | 0.29 |
|  | PLACEBO | 32.1 | 10.8 | 38 |  |  |  |  |  |  |  |  |
|  | MEDICATION | 35.4 | 11.7 | 36 |  |  |  |  |  |  |  |  |
| Animal fluency | HC | 23.5 | 4.1 | 42 | 4.37 | 0.015 | 0.006 | 0.031 | 0.601 | 0.62 | 0.50 | 0.12 |
|  | PLACEBO | 20.1 | 6.9 | 38 |  |  |  |  |  |  |  |  |
|  | MEDICATION | 20.7 | 5.6 | 35 |  |  |  |  |  |  |  |  |
| Immediate recall (Trial 1) | HC | 4.2 | 2.2 | 42 | 9.95 | <0.001 | <0.001 | 0.004 | 0.216 | 0.96 | 0.68 | 0.29 |
|  | PLACEBO | 2.1 | 2.0 | 39 |  |  |  |  |  |  |  |  |
|  | MEDICATION | 2.8 | 2.3 | 36 |  |  |  |  |  |  |  |  |
| Verbal learning (Trials 1-3) | HC | 17.5 | 5.1 | 42 | 12.6 | <0.001 | <0.001 | 0.001 | 0.218 | 1.07 | 0.79 | 0.29 |
|  | PLACEBO | 11.5 | 5.8 | 39 |  |  |  |  |  |  |  |  |
|  | MEDICATION | 13.1 | 5.8 | 36 |  |  |  |  |  |  |  |  |
| Delayed recall | HC | 6.9 | 1.6 | 42 | 14.0 | <0.001 | <0.001 | 0.001 | 0.120 | 1.15 | 0.79 | 0.36 |
|  | PLACEBO | 4.6 | 2.2 | 38 |  |  |  |  |  |  |  |  |
|  | MEDICATION | 5.3 | 1.9 | 36 |  |  |  |  |  |  |  |  |

*Overall comparison: ANOVA; pairwise comparison: Fisher’s LSD test; grey shade indicates significant result; Cohen’s *d* = 0.2 is considered a 'small' effect size, 0.5 is considered a 'medium' effect size and 0.8 a 'large' effect size.

**Supplementary Table S3.** Details of participants who were excluded from the per protocol analysis due to discontinuing their trial medication (placebo or risperidone/paliperidone).

| Case | Group | Day of stopping trial medication | Reason | New medication |
| --- | --- | --- | --- | --- |
| 212 | Placebo | 44 | Decline in functioning | Ziprasidone |
| 405 | Placebo | 48 | Participant request | Aripiprazole |
| 629 | Placebo | 104 | Lack of improvement | Aripiprazole |
| 214 | Placebo | 48 | Increased hostility | Aripiprazole |
| 209 | Placebo | 87 | Lack of improvement | Aripiprazole |
| 503 | Placebo | 104 | Increased symptoms | Aripiprazole |
| 404 | Placebo | 107 | Participant request | Aripiprazole |
| 621 | Placebo | 62 | Lack of improvement | Aripiprazole |
| 313 | Placebo | 71 | Increased symptoms | Risperidone |
| 611 | Placebo | 58 | Increased hostility | Amisulpride |
| 203 | Placebo | 111 | Lack of improvement | Paliperidone |
| 609 | Placebo | 24 | Increased suicidality | Paliperidone |
| 308 | Placebo | 97 | Increased symptoms | Amisulpride |
| 314 | Placebo | 109 | Lack of improvement | Aripiprazole |
| 402 | Placebo | 80 | Participant request | Aripiprazole |
| 506 | Placebo | 137 | Participant request | Amisulpride |
| 603 | Placebo | 182 | Increased suicidality | Quetiapine |
| 605 | Placebo | 111 | Participant request | Aripiprazole |
| 607 | Placebo | 80 | Lack of improvement | Quetiapine |
| 514 | Medication | 62 | Lack of improvement | Aripiprazole |
| 625 | Medication | 84 | Lack of improvement | Aripiprazole |
| 305 | Medication | 19 | Increased hostility | Paliperidone |
| 211 | Medication | 82 | Increased symptoms | Quetiapine |
| 504 | Medication | 66 | Participant request | None |
| 617 | Medication | 73 | Lack of improvement | Aripiprazole |
| 213 | Medication | 8 | Increased hostility | Risperidone |
| 312 | Medication | 97 | Increased suicidality | Amisulpride |
| 610 | Medication | 52 | Increased symptoms | None |
| 311 | Medication | 154 | Side-effects | None |
| 401 | Medication | 99 | Side-effects | Quetiapine |
| 107 | Medication | 106 | Increased symptoms | Quetiapine |
| 510 | Medication | 28 | Lack of improvement | Quetiapine |
| 102 | Medication | 17 | Increased symptoms | Olanzapine |
| 103 | Medication | 132 | Moved out of area | Not Applicable |
| 109 | Medication | 6 | Increased suicidality | Paliperidone |
| 206 | Medication | 108 | Lack of improvement | Olanzapine |
| 511 | Medication | 85 | Disengaged from service | Not Applicable |
| 515 | Medication | 126 | Disengaged from service | Not Applicable |
| 606 | Medication | 28 | Lack of improvement | Risperidone |
| 608 | Medication | 2 | Participant request | None |
| 612 | Medication | 43 | Participant request | Aripiprazole |
| 614 | Medication | 57 | Participant request | Quetiapine |
| 618 | Medication | 117 | Increased suicidality | Aripiprazole |
| 623 | Medication | 84 | Moved out of area | Not Applicable |
| 624 | Medication | 37 | Increased suicidality | Amisulpride |

**Supplementary Figure S1.** Reason for stopping trial medication per group.

**Supplementary Table S4.** Results of LME analysis comparing the three groups in terms of the rate of change from baseline to 6-months with patients restricted to trial completers only.

|  | P-values | | | Estimated rate of change | |  |
| --- | --- | --- | --- | --- | --- | --- |
|  | Group x time interaction | Group | Time | Coefficient | Standard Error | n |
| Digit Span: forward | 0.808 | 0.220 | <0.001 | 0.034 | 0.008 | 69 |
| Digit Span: backward | 0.549 | 0.003 | 0.032 | 0.014 | 0.007 | 69 |
| Digit Symbol-Coding | 0.016 | 0.034 | <0.001 | 0.148 | 0.040 | 65 |
| Stroop: words | 0.267 | 0.144 | 0.078 | 0.083 | 0.047 | 69 |
| Stroop: colours | 0.920 | 0.273 | 0.013 | 0.092 | 0.037 | 69 |
| Stroop: colour-word | 0.333 | 0.060 | 0.009 | 0.077 | 0.029 | 69 |
| Letter fluency | 0.364 | 0.068 | 0.007 | 0.083 | 0.030 | 69 |
| Animal fluency | 0.545 | 0.301 | 0.638 | 0.010 | 0.023 | 69 |
| Immediate paired-associate recall (Trial 1) | 0.051 | <0.001 | 0.039 | 0.022 | 0.011 | 69 |
| Total paired-associate learning (Trials 1-3) | 0.025 | 0.003 | 0.192 | 0.028 | 0.021 | 69 |
| Delayed cued recall | <0.001 | 0.005 | 0.703 | 0.003 | 0.008 | 69 |

*Note:* Placebo n=16; Medication n=11; Healthy controls n=42

**Supplementary Figure S2.** Plots of estimated trends from baseline to 6-months and associated 95% confidence intervals for the means at endpoints with FEP patients restricted to trial completers only (placebo n=16; medication n=11). Variables with an asterisk showed a significant group by time interaction.


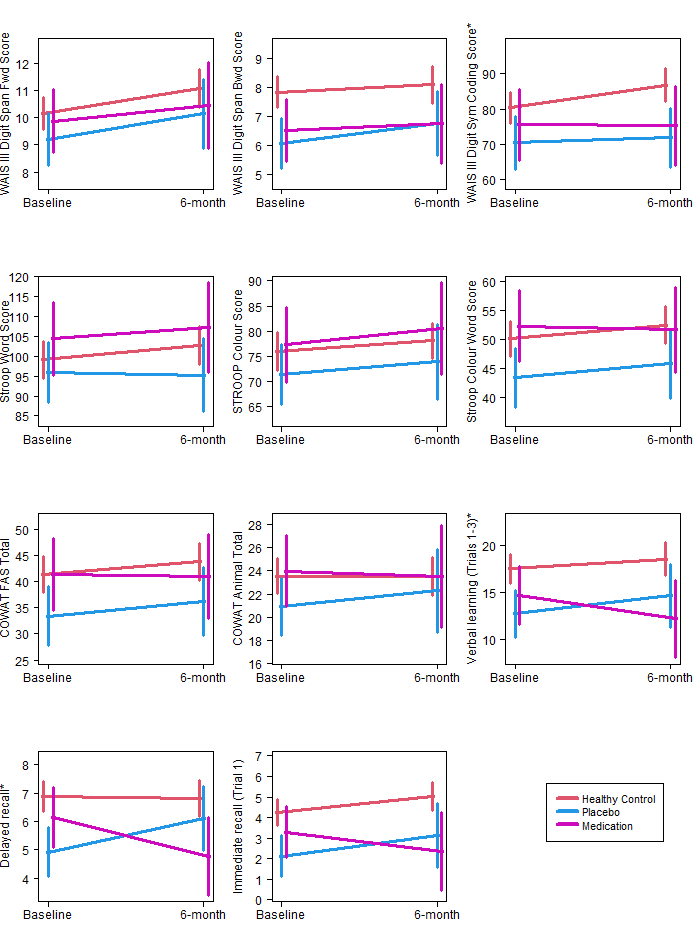


**Supplementary Table S5.** Means and SDs of the Paired-Associate Learning variables broken down by concrete and abstract word pairs within each group at baseline.

The p-values are based on linear mixed-effects model analysis comparing concrete and abstract words within each group. The results indicate that overall concrete word pairs were significantly easier to learn and recall than abstract word pairs within each group, except for Immediate recall (Trial 1) in the Placebo group.

|  |  | Medication | | | Placebo | | | HC | | |
| --- | --- | --- | --- | --- | --- | --- | --- | --- | --- | --- |
|  |  | Mean | SD | P-value | Mean | SD | P-value | Mean | SD | P-value |
| Immediate recall (Trial 1) | Concrete | 2.9 | 0.6 | <0.001 | 2.7 | 0.9 | 0.753 | 3.3 | 0.7 | 0.001 |
|  | Abstract | 2.1 | 0.7 |  | 2.7 | 1.0 |  | 2.8 | 0.7 |  |
| Verbal learning (Trials 1-3) | Concrete | 8.3 | 2.8 | <0.001 | 7.3 | 2.9 | <0.001 | 9.7 | 2.6 | <0.001 |
|  | Abstract | 4.8 | 3.4 |  | 4.2 | 3.3 |  | 7.7 | 3.2 |  |
| Delayed recall | Concrete | 3.4 | 0.8 | <0.001 | 3.1 | 1.2 | <0.001 | 3.7 | 0.7 | <0.001 |
|  | Abstract | 1.9 | 1.3 |  | 1.6 | 1.4 |  | 3.1 | 1.2 |  |

**Supplementary Table S6.** Results of LME analysis comparing the 3 groups in terms of the rate of change from baseline to week 26 assessment

|  | P-values | | | Estimated rate of change | |  |
| --- | --- | --- | --- | --- | --- | --- |
|  | Group by time interaction | Group | Time | Coefficient | S.E. | n |
| List A Total | 0.607 | <0.001 | 0.540 | -0.008 | 0.013 | 117 |

Results of LME analysis comparing the 3 groups in terms of the rate of change from baseline to week 26 assessment with patients restricted to completers only

|  | P-values | | | Estimated rate of change | |  |
| --- | --- | --- | --- | --- | --- | --- |
|  | Group by time interaction | Group | Time | Coefficient | S.E. | n |
| List A Total | 0.764 | <0.001 | 0.856 | 0.002 | 0.012 | 69 |
